# Parental Reports on Touch Screen Use in Early Childhood

**DOI:** 10.1371/journal.pone.0128338

**Published:** 2015-06-17

**Authors:** Alejandrina Cristia, Amanda Seidl

**Affiliations:** 1 Laboratoire de Sciences Cognitives et Psycholinguistique (ENS, EHESS, CNRS), Département d’Etudes Cognitives, Ecole Normale Supérieure, PSL Research University, Paris, France; 2 Speech, Language, and Hearing Sciences, Purdue University, West Lafayette, IN, USA; Goldsmiths, University of London, UNITED KINGDOM

## Abstract

Touch screens are increasingly prevalent, and anecdotal evidence suggests that young children are very drawn towards them. Yet there is little data regarding *how* young children use them. A brief online questionnaire queried over 450 French parents of infants between the ages of 5 and 40 months on their young child’s use of touch-screen technology. Parents estimated frequency of use, and further completed several checklists. Results suggest that, among respondent families, the use of touch screens is widespread in early childhood, meaning that most children have some exposure to touch screens. Among child users, certain activities are more frequently reported to be liked than others, findings that we discuss in light of current concern for children’s employment of time and the cognitive effects of passive media exposure. Additionally, these parental reports point to clear developmental trends for certain types of interactive gestures. These results contribute to the investigation of touch screen use on early development and suggest a number of considerations that should help improve the design of applications geared towards toddlers, particularly for scientific purposes.

## Introduction

Touch screens are increasingly prevalent in many societies and, according to anecdotal reports, young children are drawn to them and quickly become expert users. We aimed at moving towards a more thorough description of touch screen use in young children by collecting parental reports of infant and toddler touch screen use. This report is relevant to developmental psychology in two ways.

First, the sheer prevalence of touch-screen devices in many cultures exposes children to this technology, which may have effects on their cognitive development. The use of touch-screen devices has, up to now, mainly been studied in the context of media use in general (e.g., [1]). These investigations are marked by a debate regarding the potentially noxious effects of passive exposure to television, particularly for children under 2 years of age (e.g., [2]). In this literature, one concern relates to frequency of use, as correlational analyses suggest that for every hour children under 2 years of age spend watching TV, they spend about 50 minutes less interacting with their parents and about 20 minutes less in creative play [3]. A second recurrent finding is that evaluating the *content* of media use is paramount. For example, evidence is accumulating that certain types of television programs may promote prosocial behavior [4] whereas other programs may negatively affect executive function [5].

While it is possible that the general finding of a time trade-off will also apply to touch screen use, the modulating effects on child cognition of the content of use will almost certainly increase in importance when considering touch screens. Specifically, while television programs can sometimes engage children at a cognitive level (e.g., [6]), the content of touch screens typically also necessitates *physical* engagement; that is, if the child does not interact physically with the touch screen, nothing happens (but see discussion of the activities reportedly liked below). Touch screens can therefore rely on *contingency*, one of the features that make social contexts particularly conducive to learning (for a recent and relevant example, see [7]; and [8] for a specific discussion). In fact, some research finds salient differences in conceptual learning and imitation with set-ups involving real world objects, TV displays, and touch screens (for example, see [9–12]). It is thus not surprising that quite different opinions are being voiced regarding toddlers’ exposure to touch screens, as exemplified by somewhat optimistic statements promoting the use of interactive screen media by advisors to educators and policy makers (e.g., some associations in the United States [13]; and the French Academy of Sciences [14]).

And yet there is a dearth of research on touch screen use in early childhood [8]. Therefore, a description of touch screen use will be of interest to the many audiences (scientists, educators, parents, and policy makers) who would like to know about the cognitive impact of exposure to and use of interactive media in general, and touch screens in particular. Without such a description, we cannot decide whether or not such devices could have a place in daycares, preschools, or the home. As a first step towards addressing these key concerns, we collected very simple parental reports.

The present research project also interests a second group of readers: With the rise of touch screen use, toddler-oriented applications are increasingly common and therefore descriptions of touch screen use is relevant to those who seek to build products aimed at this population. Among these interested parties are research-oriented laboratories specializing on early development. It is very difficult to study language and cognition in children below the age of 3 years. In fact, touch screens have been used with other populations who do not readily follow explicit verbal instructions, including non-human primates, for about 20 years (for example, [15] and more recently [16]). Currently, a number of baby labs in the world are exploring the possibility of adding touch-screen applications to their experimental toolkit (for example, see [17, 18]). In this enterprise, a key concern is whether all (typically-developing) young children readily use, or can quickly acquire, the gestures required to express their responses through a touch screen. The present data was directly inspired by these goals, and our report will draw out several messages that ought to facilitate other laboratories’ search for optimal testing paradigms. Naturally, these considerations may also be of interest to designers of educational applications designed for toddlers. Although there is some literature on designing apps for older children (e.g., see an overview of the general considerations to reflect upon in [19]), there is none to our knowledge on infants and toddlers specifically.

In sum, we sought to address the following key questions:

1. How prevalent is touch screen use in infants and toddlers?
2. What activities do infants and toddlers like doing on touch screens?
3. What kinds of gestures do infants and toddlers display when interacting with touch screens?

We designed a short online questionnaire, which asked parents to report on their child’s frequency of use, activities liked, observed interactive gestures, and a number of background variables. From over 450 responses, we could have a unique quantitative perspective on the moderation of frequency of use, activities liked, and gesture prevalence with age, thus providing an initial response to questions 1–3.

Admittedly, parental checklists suffer from a number of weaknesses such as potential response biases and variability in criteria used to respond. Nonetheless, the present report makes a substantial contribution given the scarcity of research on the topic [8]. Moreover, most previous work has been conducted primarily in the United States. It is conceivable that different cultures integrate novel technologies in different ways. Although they need not be viewed as representative of their cultures, compare, for example, the somewhat divergent recommendations on media use in early childhood proposed by the American Academy of Pediatrics [2] and the French Academy of Sciences [14]. All of the data we report on was collected in France.

## Motivation and questionnaire design

Our first research question concerns how frequently infants and toddlers are exposed to and use touch screens. To address this question, we asked parents to estimate how frequently their child used a touch screen. There were two “never” options, to indicate that the child had neither touched nor seen a touch screen or, alternatively, seen but not touched one. The other options were sorted with an increase in frequency of use, ranging from less than once a month to daily use.

Our second research question concerns the kinds of activities young children reportedly like doing on touch screens. This is intimately related to the concept of “content” as it has been applied in research on television. Parents could check one or more of 4 options: Looking at photographs, watching videos, playing with apps based on sound-image associations, and playing with puzzles. Clearly, this is far from a comprehensive list. We tried to keep the questionnaire short, and only included this small set based on informal parental surveys (which indicated these as fairly common activities) as well as considerations of the types of activities that could later be used to collect cognitive data. Parents could also check “other” and complete activities that they considered their child liked (including applications that may not have fit into any of our categories) with their own words.

Naturally, the activities children may exploit could vary as a function of a number of variables, possibly the frequency of touch screen use, and most saliently age. Indeed, whereas children of all ages are probably able to engage in passive activities like watching videos, more complex activities should be within the reach of only older or more advanced children; for example, puzzles will more likely be enjoyable for toddlers who have the motoric control to produce the fine gestures required to control the puzzle pieces.

At this point we turn to our third research question. With it, we wanted to venture into the more precise question of *how* infants and toddlers manipulate touch screens. A number of interactive gestures are typically part of adult touch screen users’ gestural repertoire. We asked parents to report whether they had observed their child producing one or more from a list of common gestures, which can be ordered in terms of dexterity required to master them, from the simplest one (tapping) to the most complex (spreading and pinching actions, which require accurate control and coordination of two fingers). In addition, we also asked parents whether they had observed their child banging on the screen (a gesture that is not typical in adults’ interactions with touch screens, nor is it required of any app that we know).

We expected that the likelihood of a given gesture being present would depend on the child’s development and the amount of practice with touch screens (the latter gauged through frequency of use). As noted above, we collected age information, which is a good proxy for development, but we additionally hoped to shed light on the potential connection between the interactive gestures a child used on a screen and other gestures that he/she may display, which may be a more direct index of individual development. We therefore asked parents about a number of communicative gestures (namely, pointing, waving, nodding) as well as grasping gestures involved in tool use. For the latter, parents answered three yes/no questions, assessing whether their child could hold a bottle, use a fork, and draw. We expected that both communicative and tool gestures would be increasingly frequent with age. Additionally, we expected to find that children whose parents reported them to use communicative and/or tool gestures might also (reportedly) display more complex interactive gestures with their touch screens.

In addition to the variables noted above and which directly related to our research questions, we also collected some ancillary data, such as what type of hardware the child used. The complete questionnaire is available in the Appendix.

## Participants

Publicly available, voluntary questionnaires are exempt from revision by an ethical committee in France. We sent a link to the questionnaire to a randomly selected sample of 1000 parents whose infants were currently between 5 and 40 months of age and had participated at the LSCP Baby lab at least once, and for whom we had an email address. Respondents were able to forward the link to others if they desired to do so.

Data collection began on December 2, 2013; for the purposes of this report it was closed on December 10, 2013. By then, 505 responses had been recorded, of which 478 were complete and could be analyzed further. Of these, 25 responses were excluded because the child was older than our established maximum age (40 months). Among the remaining 453 records, 229 were reports on female infants and toddlers. Median age, in days, was 778 (about 2 years and 2 months), 25% quantile 533, 75% quantile 1015, and range 156–1216. Six infants had received a diagnosis that could lead to developmental delays. Given their small number, and in the interest of establishing general facts, we did not remove their data from subsequent analyses.

Properly anonymized data and ancillary files (analyses script, information to participating families), for this and additional studies provided as supplementary materials, are available from https://osf.io/2m8iv/files/.

## Analyses

All analyses were performed using R [20]. Descriptive analyses were prioritized. When necessary, logistic models were used to assess to what extent the answers to our research questions varied as a function of certain moderators, as follows.

Age (in days) was centered and treated as a numeric variable. Although there is in principle no reason to assume that the type of changes with age that we might observe here will not be linear, we inspected the data in additional ways, namely by splitting infants on the basis of age deciles through stacked barplots.

Frequency of use was converted into a factor. This allowed us to fit a separate estimate for each level, and thus avoid the assumption that effects increased linearly (since the intervals we used where not linear: “less than once a month” is not the same number of units away from “less than once a week”, as the latter is from “less than once a day”).

As noted above, we also collected parental responses on whether the child used communicative gestures (3 questions) and engaged in certain types of tool use (3 questions). To simplify the data structure, we created two Boolean variables, “communicative” and “tool”, which were zero if the parent answered “none” in all three relevant questions.

Whenever multiple questions were integrated into a single regression model, the record identity was declared as a random factor. In all regressions, Type II Analysis of Deviance were used (from the package car [21]) to assess statistical significance.

Given the exploratory nature of the present study, several regressions were run. To focus on the most salient effects, the alpha significance level was set at.001 prior to analyses.

## Results

### Research question 1: Reported prevalence and frequency of use

**Fig 1:**
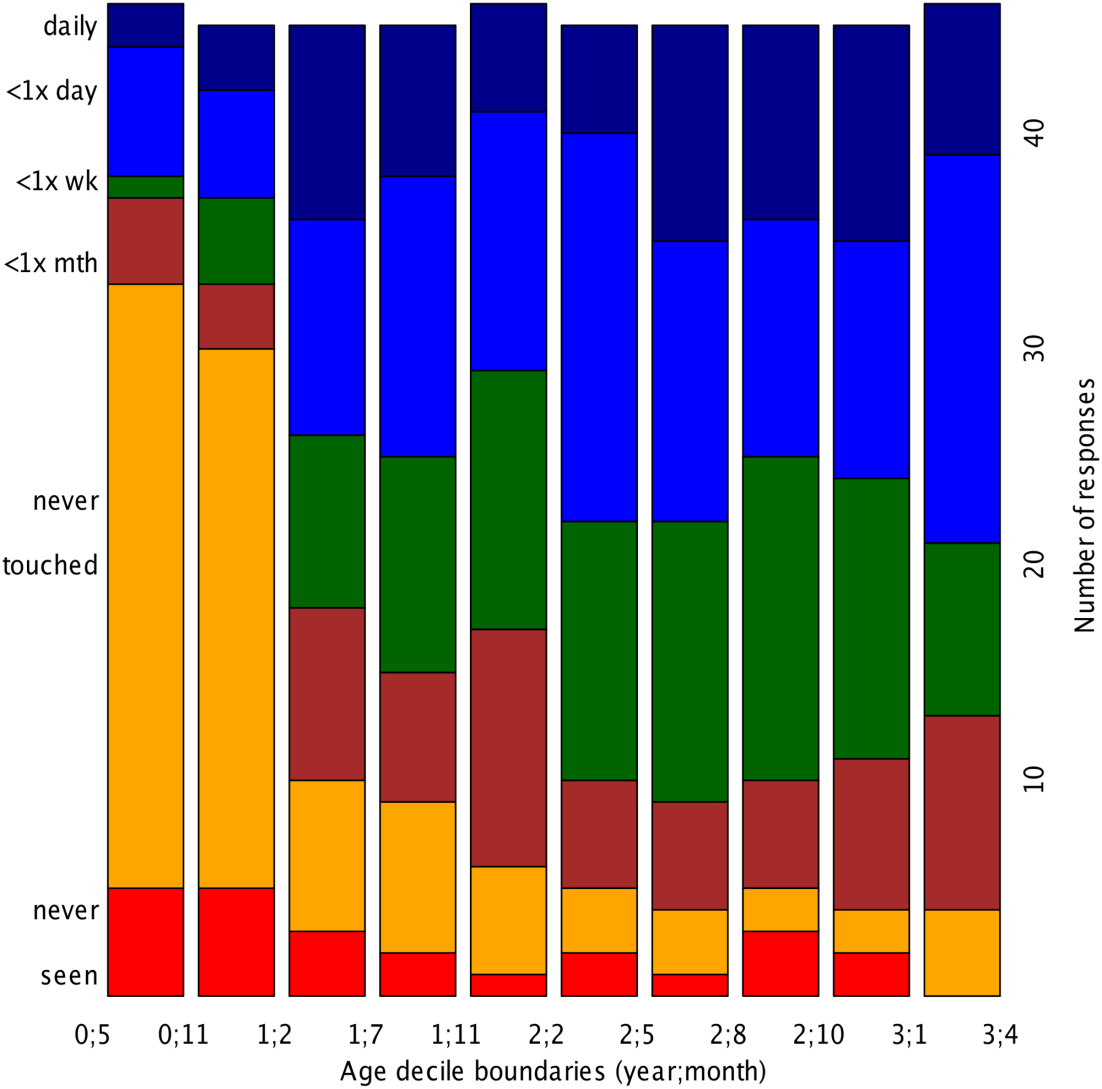
Frequency of use by age. Data is grouped in deciles to even the number of infants in each frequency of use category; “<1×” stands for “less than once a”; “wk” stands for “week” and “mth” for “month”.

The stacked histograms in Fig 1 represent the number of parents who selected a given response among the options indicating frequency of use. The abscissa is given in deciles to permit an easier visual inspection, as the number of infants varied greatly as a function of age.

This figure clearly shows that the proportion of children who have never used a touch screen decreases dramatically with age. Overall, 24% of the sample (N = 110 infants) were said to have never used a touch screen, and this was more common among younger infants. Indeed, among the first two deciles (roughly 5 to 12 months, and 12 to 14 months), non-users make up nearly two-thirds of responses (33/46 and 30/45, respectively). This proportion of non-users per age decile then drops to one third (10/45) and stabilizes at 9–13% between the fifth and tenth deciles (roughly from 2 years of age onwards).

**Fig 2:**
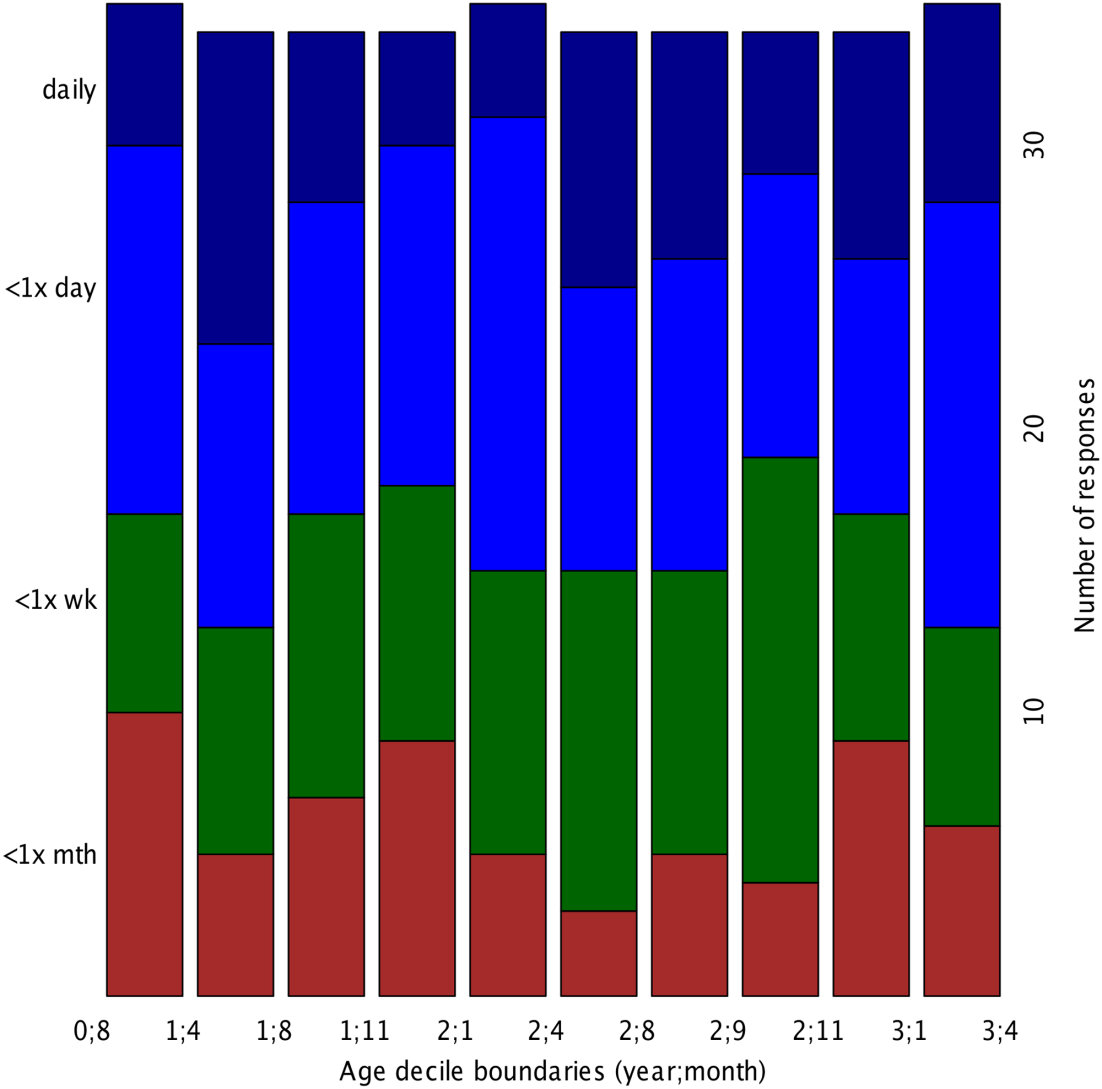
Frequency of use by age (in year;month) among children reported to have used touch screens in the past. Data is grouped in deciles to even the number of infants in each category. As in Fig 1, “<1×” stands for “less than once a”; “wk” stands for “week” and “mth” for “month”.

Interestingly, frequency of use does not appear to relate to age in any straightforward way. This is clearer in Fig 2, which represents the number of responses for the different frequency estimations by age decile among users. Notice that for this graph we recalculated the age deciles on the basis of the age distribution among “users”, that is, infants with reported frequency of use “less than a month” or more frequently than that (N = 345). The observation that frequency does not vary with age was checked through parametric statistics, and neither a regression predicting age in days from the frequency of use category, nor a *χ* ^2^ on the co-occurrence between decile and frequency of use category, revealed a significant relationship between age and frequency of use.

### Research question 2: Activities reportedly liked

As explained previously, parents were asked what activities their child liked doing, and they could check zero or more out of 4 options (looking at photos, watching videos, playing with sound-image association baby apps, solving puzzles); they could also type in other answers. Collapsing across ages and frequencies of use, the most common activity that caregivers checked was looking at photos (78%) with videos being a close second (68%). About half of the parents reported that their child enjoyed baby apps, and only a quarter said so for puzzles. Some of the respondents typed in activities related to music (4% of users; e.g., baby piano), other types of games (5% of users), or some kind of non-directed interaction (5% of users; such as wildly touching the screen).

Binomial logistic regressions were used to predict the likelihood of an activity being chosen from the activity identity, the frequency of use (declared as a factor), and age. The sound-image app was taken as the baseline activity, given that it was present in about half of the respondents. The formula was: glm(presence = activityID * age.c * frequency.cat + (1/record), data = table, family = “binomial”), were age.c stands for centered age in days and frequency.cat for a categorial version of the “frequency of use” responses. This initial model revealed significant main effects of activity identity [*χ* ^2^ (3) = 247.018], frequency of use [*χ* ^2^ (3) = 33.326], and age [*χ* ^2^ (1) = 31.835], as well as a marginal interaction between activity identity and age [*χ* ^2^ (3) = 14.666, *p* = .002].

Since frequency of use did not interact with activity identity, we discuss the main effect of frequency of use without separating the different activity types, as follows. The main effect of frequency of use emerged because parents of more frequent users were more likely to report that their child enjoyed an activity. Indeed, among children reported to use tablets in the lowest frequency category (less than once a month), the proportion of “yes” collapsing across all activity categories was 44%. This went up for each of the following frequency categories: for less than once a week, the proportion of activity boxes checked was 51%; for less than once a day, 60%, and for the highest frequency category, daily, it was 64%.

**Fig 3:**
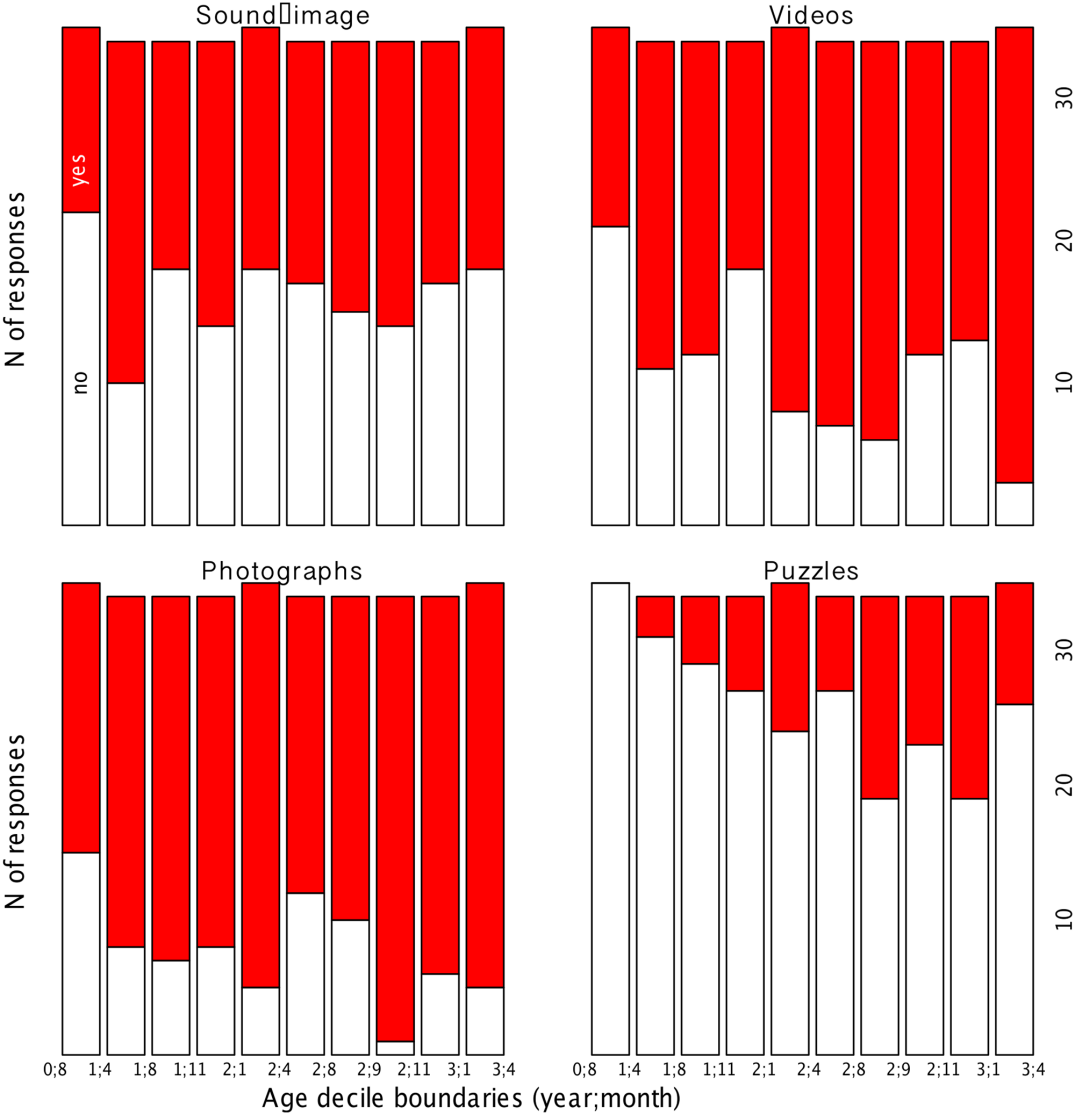
Activity reportedly liked by age decile. Caregivers’ report of whether their child enjoyed an activity (red) or not (white), grouping children in ten equally-sized age groups. The limits of some of the age groups (in year;month) are given in the x-axis (see Fig 2 for full list).

Since the interaction between activity identity and age renders an interpretation of the main effects of these two variables difficult, we carried out regressions on each activity separately. Given that frequency of use did not interact with activity identity or age, it was not included in the follow-up regressions. The results of the four binomial regressions (one for each activity in the questionnaire) are summarized in Table 1. Putting these results together with Fig 3, we can offer a tentative interpretation of both the main logistic regression effects and the interaction. It appears that, overall, most children reportedly like viewing photographs and videos, and that this preference increases with age in the case of videos. In contrast, about half of the children at any age are said to like sound-image apps, with no significant developmental differences. Few children are said to like puzzles, although this proportion increases dramatically with age. Indeed, the main effect of age likely captured the fact that “yes” responses are, overall, more likely the older the child is.

**Table 1:** Effects of age on activity reportedly liked.

Activity identity	*χ* ^2^ (1)	p
Sound-image	0.153	.700
Viewing photographs	6.625	.020
Watching videos	15.275	< .001*
Puzzle-solving	25.07	< .001*

Results from logistic regressions attempting to predict whether parents report that their child likes an activity (1) or not (0) from the child’s age (Type II Analysis of Deviance).

* indicates significant given our pre-established alpha level.

We carried out one further analysis. As expressed in our Motivation section, we specifically predicted that puzzles would be more enjoyable for children who had the ability to use the gestures required by puzzle apps. It should be pointed out that we do not know that *all* current puzzle apps require advanced gestures, only that many puzzle apps in preliminary investigations by the authors did require these gestures. Specifically, the puzzle apps geared at young children that we tested (in preliminary work) involved press and drag, with the participant having to press on a puzzle piece in the periphery, move it to a precise target area, and lift their finger only when the piece is correctly aligned. Based on our experience, we expected more frequent “yes” responses for children reported to have the press and drag gesture, controlling for age; and we expect no relationship with other gestures—for instance, with the presence of the banging gesture. We chose banging because it is the most extremely different gesture, probably typical of low motoric control (see the next subsection for evidence supporting this assumption). Therefore, the response to the ‘banging’ category makes it a good control for parents simply being more inclined to respond “yes” to all questions. Testing this highly specific prediction also allows us to indirectly validate the specificity of parental responses. Specifically, we fitted a logistic regression with the formula glm(puzzle = age.c + pressAndDrag + bang, data = table, family = “binomial”). A Type-II Analysis of Deviance revealed that, even when we had controlled for age [*χ* ^2^ (1) = 11.032] and a yes-bias (represented in a “yes” response to the banging gesture [*χ* ^2^ (1) = 3.240]), the presence of press and drag was significantly related to the responses to the puzzle item [*χ* ^2^ (1) = 23.042].

In short, these results suggest that some activities are enjoyed (according to parental report) more than others, with photograph and video viewing being more frequently selected as enjoyable than sound-image apps and puzzle apps. Additionally, parents of older children are more likely to report that their child enjoys an activity and this is more marked in the case of videos and puzzles. Interestingly, frequency of use did not modulate the effects of age, although positive answers were more prevalent among parents of children reported to employ touch screens more frequently. Finally, we found specific relationships between motor dexterity in one specific gesture (press and drag) and one touch-screen activity reportedly enjoyed (puzzle).

### Question 3: Interactive gestures

Averaging over all ages and frequencies of use, the proportion of child users reported to display each interactive gesture was as follows:

- bang on screen (with an open hand): 16%
- tap (quick one finger touch): 71%
- flick (quickly brush surface with a fingertip, as if turning a book page): 68%
- press (touch and hold for an extended period of time): 36%
- press and drag (touch with one finger and while holding down, move finger slowly): 41%
- swipe (touch with multiple fingers and while holding down, move them slowly): 20%
- pinch (touch surface with two fingers and move them together, e.g., to zoom out while viewing a photograph): 10%
- spread (touch surface with two fingers and move them apart, e.g., to zoom in while viewing photograph): 15%

**Fig 4:**
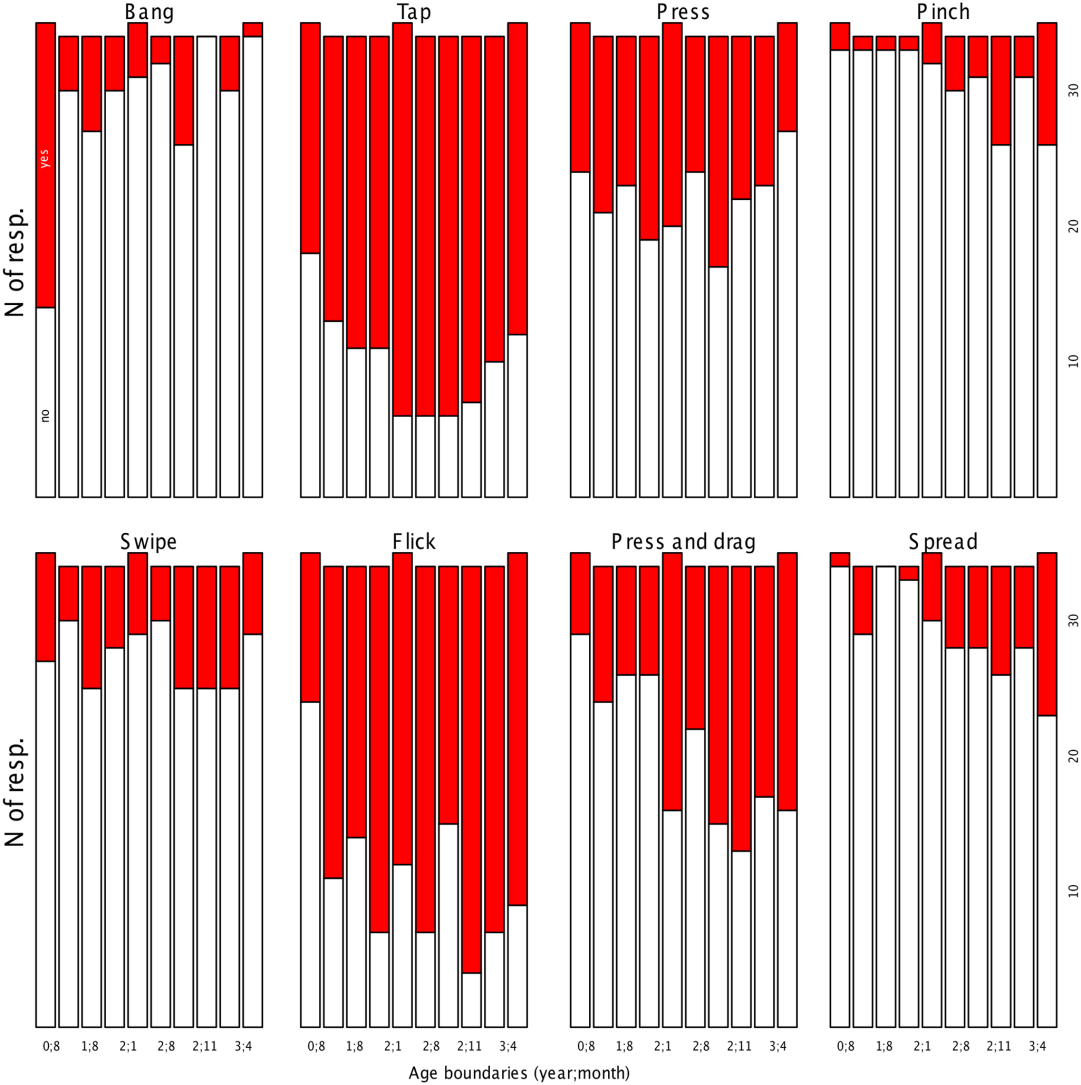
Gestures displayed during interaction with a touch screen by age decile. Caregivers’ report of whether their child displayed an interactive gesture (red) or not (white), grouping children in ten equally-sized age groups. The limits of some of the age groups (in year;month) are given in the x-axis (see Fig 2 for full list).

However, as explained above, it is likely that the presence of certain gestures, particularly pinch and spread, depends on the child’s age. In fact, inspection of the distribution of responses for each gesture and age decile (Fig 4) suggested important changes with age.

As explained in the Motivation section, we further hoped to shed light on the relationship between gestures used when interacting with touch screens and gestures the child might display in their everyday life, either for the purposes of communication or when handling tools. We planned to carry out a logistic regression including all of these predictors. Before proceeding, since the communicative and tool use gesture items we used are not part of a standardized test, we first checked that these variables showed a reasonable distribution, with frequency reportedly increasing with age and reasonable variation across individuals.

Inspection of the both communicative and tool variables revealed that there was not a great deal of variation across children, since parents of most children reported them to produce at least one, and frequently all communicative or tool-use gestures. Indeed, only 12 and 13 children had a “no” for communicative and tool gestures, respectively. Therefore, we did not include these factors as predictors in our regression.

As when investigating the predictors for activities, we created a dataset including all gestures as repeated measures from each child, with a binomial dependent measure (1 = reported present, 0 = otherwise). We declared as fixed effects the gesture identity, the frequency of use, age, and their two-way interactions. The gesture bang was declared as baseline (alphabetic ordering). The formula was: glm(presence = gestureID*age.c + gestureID*frequency + (1/record), family = “binomial”). Readers may wonder whether we should not have tried to fit non-linear patterns with age, since some of the plots in Fig 4 appear to show u-shaped changes with age. We did entertain this possibility. However, a polynomial regression with squared and cubed age regressors (in interaction with gesture identity) did not reveal contributions that met our pre-established alpha. Therefore, we concentrate on the regression considering only linear effects of age.

All the main effects surpassed our pre-established alpha: gesture identity [*χ* ^2^ (7) = 638.18], age [*χ* ^2^ (1) = 21.27], and frequency of use [*χ* ^2^ (3) = 50.01]. There was also an interaction significant at the.001 level, involving gesture identity and age *χ* ^2^ (7) = 97.21].

We interpret first the main effect that was not involved in an interaction, namely frequency of use. The main effect of frequency of use emerged because caregivers who reported their child to use touch screens more frequently also checked more gestures: For children using touch screens less than once a month, an average of 24% of the boxes were checked; for those with frequencies of less than once a week, 33%; those using touch screens less than once a day 37%; and for every day frequency users, 42%.

Since gesture identity interacted with age, we carried out separate regressions for each gesture type. The formula used was glm(gesture = age.c, family = “binomial”). As apparent from Table 2 and Fig 4, overall, the proportion of “yes” responses increases with age. The change is more dramatic in press and drag, but it is visible also for pinch and spread. Other gestures appear more unstable in this decile-based view, even though in most datasets age was significant (Table 2). Notice, finally, that the frequency of banging *decreases* with age.

**Table 2:** Effects of age on interactive gestures reportedly used.

Gesture identity	*χ* ^2^ (1)	p
Bang	36.35	< .001*
Tap	6.939	0.008
Flick	15.784	< .001*
Pinch	13.455	< .001*
Press	0.398	0.528
Press and drag	26.272	< .001*
Spread	19.222	< .001*
Swipe	0.201	0.654

Results from logistic regressions attempting to predict whether parents report that their child, when interacting with the touch screen, has used a given gesture (1) or not (0) from the child’s age (Type II Analysis of Deviance).

* indicates significant given our pre-established alpha level.

## Discussion

We discuss our results regarding the three key research questions laid out in the introduction, with a view to informing two communities. On the one hand, our results interest those who are concerned with the potential effects of electronic media on child development, including parents, educators, and psychologists. On the other hand, we hope to inform those who intend to use touch screens to reach a child audience, most saliently experimentalists desiring to expand their developmental toolkit. We will attempt to draw insights both from our own data, and, when possible, from previous reports on children growing up in the US.

### Question 1: Prevalence and frequency of use

Our first research question focused on the prevalence and frequency of touch screen use. It is possible to discuss this question using not only our data, but also drawing some cross-national comparisons with the Common Sense 2013 report [22], which included several similar items asked of American parents of children 0–8 years of age. Results from that report are provided collapsing across all age groups, and sometimes additionally focusing on children younger than two years of age. Given that results are very likely to change radically when children enter the educational system (because their schedule changes), we do not compare our results for 5–40 months with theirs for 0–8 years, but rather compare their 0–2 years with our 5–24 months.

Use of touch-screen or mobile devices appears much more wide spread in our sample than in theirs. Indeed, whereas 67% of children younger than two years of age had never used mobile media according to the Common Sense Media 2013 report, only 42% of the parents of children aged 5–24 months who completed our questionnaire reported that their child had never used touch-screen devices before. This important difference could be due to a number of reasons. As mentioned above, they focused on American parents whereas we collected data primarily from French residents, and perhaps partially this difference reflects a divergence in habits.

However, we believe that this difference is primarily, or perhaps solely, due to several methodological differences between the Common Sense data and ours. First, we just mentioned that their sample includes children 0–5 months of age, whereas ours does not, and it is likely that such young infants have low proportions of use. Second, their sample was recruited over the phone to produce a nationally-representative sampling, whereas we used email to invite former participants at the LSCP baby lab to respond to an online questionnaire. Several aspects of the latter recruitment and participation method could lead to a biased sample in terms of prevalence of touch screen use. Indeed, the LSCP baby lab is located in the center of Paris, and thus more accessible to families having a higher socio-economic status (SES), and furthermore, participants are not paid for their participation. Thus, former participants tend to have higher SES than average. It is likely that oversampling from high SES will lead to higher reports of touch screen use; for instance, in the Common Sense 2013 report, only 22% of low SES families of children aged 0–8 years had a mobile device at home, whereas 63% of higher-SES families had one. Similarly, recruiting parents over email and asking them to respond online may have biased our sample to more technologically oriented parents, who may also be more prone to having touch-screen devices and/or letting their child play with one.

Although at present we cannot demonstrate that these methodological differences explain such a large (25 percentage point) difference in prevalence, some preliminary data from a second version of our questionnaire indicates that this is indeed the case. We shared a second version of our questionnaire with both American and French parents that had participated in our respective baby labs. The number of responses is too small to discuss their results extensively, but we would like to point out that prevalence is much more similar using these two better SES-matched samples, yielding 38% for the American respondents and 46% for the French ones. Further information (including the raw data, analysis scripts, and a fuller report of results) can be found in the Supplementary Materials https://osf.io/2m8iv/files/.

While the use of devices is more widespread in our sample than in previously published American ones, our French children do not appear to use such devices more *frequently* than children in the Common Sense 2013 sample. Indeed, 23% of the children 0–2 years who had ever used mobile devices did so daily according to the Common Sense report, versus 21% in our data. As for lower frequencies, we separated ‘less than once a day’, ‘less than once a week’ and ‘less than once a month’, whereas Common Sense only separates weekly versus less frequent than that. If we consider their ‘weekly’ to map onto our ‘less than once a day’, the overall percentages are uncannily similar. They report that, among children who have used a mobile device (i.e., the parent did not respond ‘never’) 31% responded weekly versus 32% who chose ‘less than once a day’ in our sample. The remaining 46% of the Common Sense sample used touch-screen devices less frequently than weekly, compared to 48% of our sample (25% ‘less than once a week’ + 23% ‘less than once a month’). Additionally, we would like to point out that frequency of use does not increase with age in our sample, which goes up to 40 months of age (3 years, 4 months). This information is unfortunately not provided in the Common Sense report.

What are the implications of these prevalence and frequency results for the two communities that we are seeking to inform? Even though we have found some differences in overall prevalence (which may well be due to sampling variation), it appears that both French and American children are indeed exposed to touch screens, according to parental reports. This reinforces the popular impression that research is needed to understand the potential effects of using such technology with children. However, we believe that both our (and the American results) on frequency of use suggest that measuring these effects through individual variation in the population will be challenging. Indeed, notice that nearly half of the children in both samples used a touch-screen device less than once a week. Although we do not know for how long they engage in touch screen use in that one weekly occasion, it is unlikely that this would result in a significant displacement of or competition with other activities (such as creative play, interaction with parents, etc.) As a result, the real-life effects of touch screen use are bound to be so small and variable as to escape detection, except if extraordinarily large samples are used. A large sample would also be necessary to measure effects even in the highest category of use (at least once a day) because only 18–23% of all users younger than age 2 belong to this category. In other words, only 6% of all children 0–2 years of age in the American report [22], and 12% of our sample fall into this category.

While these results may assuage concerns related to ways in which touch screens may be interfering with child development, they also indicate that people who are interested in using touch screens to reach out to child audiences will have a hard time doing so. Indeed, should child-directed apps be created (scientific or otherwise), it appears that few children would have access to them. French children seem, at first blush, to lend themselves more readily to becoming a target audience given that the proportion of users is higher than in the American reports, but we remind readers that the prevalence numbers may be inflated due to oversampling from high SES populations and/or technologically oriented ones. Particularly when the goal is to study child development, then such a sampling bias is problematic because ensuing results would not reflect average performance, but that of children growing up in a very specific kind of environment. Our frequency results further indicate that most children are provided with the opportunity to use touch screens less than once per week. This means that high-density sampling of child performance over time would be unlikely, simply because too few children would be using a touch screen frequently enough to provide sufficient data. Moreover, as we will explain below, any app would have to compete with other activities infants and toddlers like doing on touch screens (according to our parental reports).

### Question 2: Activities reportedly liked

Our second research question concerned content. Although some of our questions are similar to those asked by Common Sense, their data are not broken down by age, and in any case content does not seem to be broken down into categories within mobile devices (but only for TV). Therefore, since we cannot at this point make any meaningful comparisons between our data and theirs, we concentrate on ours. We would like to start by recalling for readers that we found a main effect of frequency that did not interact with the type of activity; thus, caregivers tended to check more boxes when their child used touch screens more frequently, with no clear differences across activity types. The main effect could indicate that children who are more avid users diversify their interests in variable ways and they thus end up liking more things; or it could mean that caregivers can more easily observe the enjoyment, because it occurs more frequently. What is more telling is the lack of an interaction with activity type. Insofar as null results can be interpreted, we would like to draw an implication for those considering building apps for children. Specifically, the lack of an interaction could indicate that it is not the case that an app will be only appropriate to very heavy users. Instead, it is possible that all reasonably easy and interesting activities could appeal to all children, regardless of how frequently they use touch screens.

Next, we discuss specific activities. Surprisingly, we found that watching videos, an activity that may not immediately exploit the interactive capabilities of touch-screen devices, is very common. On average across all children, about 68% were reported to enjoy viewing videos, although percentages increased with age, from about 57% in the youngest three age deciles to 73% in the oldest three (see Fig 3 for a break down by age deciles, or download the data from https://osf.io/2m8iv/files/ to gain an even fuller picture). It is, however, entirely plausible that children do exploit the touch screen’s functionalities in this case, for instance by selecting the videos themselves. Additionally, we did not enquire as to what kind of video content children viewed. What we can say with certainty is that future questionnaires interested in the effects of video viewing should take into account videos presented via touch screen, in which case parents should be asked not only about the contents of the videos but also whether it is the child who selects them.

Despite its popularity, watching videos is not, in fact, the top ranking activity. Interestingly, viewing photos was the most common activity, with an average of 78% of respondents checking this option in the question enquiring about activities enjoyed by their child. There is a slight trend towards an increase with age, with an average 71% for the first three age deciles and 88% for the last three. Since young children reportedly enjoy looking at photos with caregivers, this activity could create a context for rich language interaction between parents and children, because the former can point out salient people in the child’s life, remind the child of certain events, etc. In other words, perhaps even more than books (which provide a content that is external and potentially irrelevant to both caregiver and child), photographs are necessarily socially relevant stimuli, at least to the parent. Future work could further enquire into this possibility, or employ observations to assess how parents and children interact with each other in the context of photo viewing on touch-screen devices.

We additionally asked about two game-like activities, baby apps that associated an image with a sound and puzzles. We found that sound-image apps were reported to be enjoyed by about half of the respondents, with no change with age. In contrast, puzzles were used more rarely, with only 8% of caregivers in the first three age deciles reporting that their child enjoyed puzzles. This percentage increased markedly with age, reaching about 34% for the last three age deciles. We further found a highly specific relationship, indicating a correlation between the reported ability to press and drag, on the one hand, and reportedly enjoying puzzles on the other, after controlling for age. These results have salient implications for those interested in building apps to study cognition, action, and perception in young children. While building a challenging app, such as one requiring displacement of items in the screen, may not entail that one’s results are biased depending on whether the child is a heavy or a light user, it does appear to limit one’s options in terms of ages. Put differently, simple apps with image-sound correspondences may be more appropriate tools to study a range of cognitive skills in children between 4 and 40 months, than what appears at first blush like a more fun and active setup involving manipulation of on-screen objects. Although, as mentioned above, the results of our second questionnaire are too small to warrant full discussion, we would like to invite readers to read the report provided in the Supplementary Materials https://osf.io/2m8iv/files/ to find out about prevalence of other types of activities (e.g., music, speaking, etc.)

### Question 3: Interactive gestures

Turning now to our third and final question, regarding interactive gestures, we explore an even less studied topic, whose results will interest primarily those thinking of building apps for young children. Our results reveal that tap was the most frequent gesture reported to be produced by young children (checked by 71% of respondents). A closely related gesture, flick, has a similar average frequency of 68%. Given their similarities in structure and prevalence, we do not discuss them separately. Inspection of Fig 4 suggests a non-linear, u-shaped change with age for both, with greatest proportions of ‘yes’ responses in the center deciles. Given that neither linear nor polynomial age regressors met our pre-established alpha level, we refrain from offering an interpretation of whether and why changes in tap presence may occur with age. Moreover, even if there are changes with age, it is still the case that tapping/flicking is, according to parental report, the most prevalent gesture, making it a good candidate for apps that are directed to young children.

The second most common pair of related gestures are press, which is checked by about 36% of parents of users, and press and drag, 41%—although the latter is rare among young infants and becomes more prevalent with age. We suggest that these two gestures are not promising for a child-directed app because they are so variable across ages and less common (according to our parental reports). In other words, apps built such that the user is required to use one or both of these gestures might be unusable by about two thirds of children.

An interesting case is that of swipe, pinch, and spread, given that over three quarters of respondents said their child enjoyed viewing photos on the touch screen. When viewing photos, swiping is usually the gesture allowing to move on to subsequent or previous photos in the gallery; and yet swiping was not very commonly checked by caregivers. Similarly, pinching and spreading allow zooming in and out, yet they are vanishingly rare early on in the present parental reports.

For the latter gestures, the explanation may lie in the level of dexterity required to control the gesture, since both pinching and spreading necessitate separate control of specific fingers in addition to a complex flexion motion. Not surprisingly, pinching becomes more prevalent with age, probably as children learn to exercise this control. Conversely, banging, a very simple and ballistic gesture, is present early on but disappears with age, possibly as the child learns to interact more appropriately.

Finally, we sought to investigate whether tool use and communicative gestures were predictors of interactive gesture prevalence. Unfortunately, we did not have enough variation in the population to assess meaningful relationships. We think that future work could address this question by adapting the items to the child’s age, asking about less and more complex benchmarks relative to the reported age of the child.

### Limitations and open questions

In the previous subsections, we have identified a number of limitations of the present data, as well as pressing questions that future research should address. The most salient limitation of our data pertains to sampling, which limits the generalizability of our results. Specifically, we found relatively inflated percentages of use when comparing with an American sample that better controlled for SES. However, notice that we did not find differences in the distribution of frequency of use across the two samples, which may indicate that SES does not play a major role in this case. Even so, we would like to point out that both France and USA are relatively wealthy countries, and thus it would be of interest to extend the investigation in the prevalence and use of touch screens to other cultures with more variability in SES.

We believe such an investigation would be most informative for the questions of frequency of use and displacement of competing activities, but we have no reason to believe that the activities reportedly enjoyed or the interactive gestures reportedly employed (the two other aspects on which our data bear) would vary a great deal with the home culture. We deduce this from the fact that we saw no interactions of activity type or gesture type with frequency of use, and only considerable interactions with age. In other words, although our data are certainly limited, it appears that at least some activities are reportedly enjoyed by all child users, and similarly some gestures are reportedly prevalent in all child users, and when this is not the case it may be due more to maturation of the motoric skills required by the activity or gesture than to the content of the activity itself. Preliminary evidence supporting these statements can be found in the Supplementary Materials.

At this point a second limitation becomes evident, and that is that a very restricted selection of activities were offered as choices to the parents. We believe future work would provide even stronger insights by both asking for more specific information on the categories we included (e.g., splitting up video-watching more finely into kinds of videos), and by adding other options (in particular, talking, reading/literacy, and musical activities were common options entered by parents as free text; see further information in the Supplementary Materials). Touch screens are very rich in terms of the types of content that can be available, and thus it will be of descriptive interest to provide a richer picture of what children actually do with a potentially limitless set of apps.

This brings us to the third limitation that we would like to underline, which is the fact that all of our conclusions are based on parental report. We cannot at present know to what extent such reports are representative of reality, not only because parents may have beliefs as to what is appropriate to report and what is not (and more generally, what activities they allow their child to engage in), but also because they may be unable to do so. For instance, we asked caregivers to assess whether their child had ever displayed a certain array of gestures, and parents may vary both in their recollection of the actual gestures and their separate recognition of the different types, and they may additionally vary on their criteria of whether a gesture was present or not. Similarly, parents may vary in what they view to be a “puzzle”. Such a problem cannot be addressed through questionnaires, but would be more appropriately addressed through observation. We carried out two small-scale studies to this end, whose results are available as Supplementary Materials (downloadable from https://osf.io/2m8iv/files/). These studies suggest that, by and large, our conclusions above are supported, although the prevalence of some gestures may be under-estimated by parents and/or easily taught. For example, in a small-scale observation, we focused on swiping and press, and would demonstrate these affordances to the child if he/she did not exhibit it already. Nearly all children could control or learn these gestures within a few minutes of touch screen use.

## Conclusions

We have reported on a parental questionnaire asking about the prevalence of touch screen use among children, the frequency with which they use such technology, the kinds of activities they enjoy, and finally the gestures they employ to interact with such tools. This has allowed us to identify some potential differences between our largely Parisian, high-SES sample and an extant, American-representative sample (in terms of prevalence) and some similarities (in terms of frequency of use). Findings regarding activities invite further research as parents report that photograph viewing is a well-liked activity among young children, which may represent an opportune setting for caregiver-child interactions. Additionally, video-viewing is very common, and this latter finding should fuel reflection for those concerned that passive watching may have detrimental effects on cognitive development. Finally, for the benefit of researchers interested in developing apps for young children, we have identified more and less promising activities. Specifically, we found that image-sound co-occurrence apps are reportedly liked by a majority of child users, with few age changes, whereas puzzles are not as popular according to these parental reports. Further, we found that, although we do not know the cause of these differences, among interactive gestures, tapping is widespread and relatively stable across ages, while more complex gestures such as pressing and dragging were less frequently reported.

## Appendix: Questionnaire translated into English

It is difficult to know what babies know; that is why our baby lab is always looking for new, smart ways of finding out. We are now developing a touch screen-based application to measure language processing in children aged 5 months to 40 months. To make this application adaptable to everyone (older and younger babies, those who are ‘smartphone pros’ and those who have never seen one), we need your help.

Please fill in the following questions as honestly as possible. Your responses are completely anonymous.

### Page 1 (Use frequency)

How often does your child use or see touch-screen technology?

0: never seen or touched; there are no tablets or smartphones at home

1: never used such technology, although he/she may have seen me and other family members interact with one

2: has occasionally used such technology, but not more than once a month

3: occasionally uses such technology, but not more than once a week

4: regularly uses such technology, but not more than once a day

5: uses such technology every day

### Page 2 (skipped if never used; Contents of use)

What does your child like doing with the touch-screen device? Check all that apply

Look at photographs

Watch videos

Complete puzzles

Use baby applications where there are images, for instance of a cow, which make sounds when pressed (e.g., moo or hear the name ‘cow’)

Others, please describe

### Page 3 (skipped if never used; Gestures)

Relying on your memory, have you seen your child perform the following gestures?

- bang on screen (with an open hand)
- tap (quick one finger touch)
- flick (quickly brush surface with a fingertip, as if turning a book page)
- press (touch and hold for an extended period of time)
- press and drag (touch with one finger and while holding down, move finger slowly)
- swipe (touch with multiple fingers and while holding down, move them slowly)
- pinch (touch surface with two fingers and move them together, e.g., to zoom out while viewing a photograph)
- spread (touch surface with two fingers and move them apart, e.g., to zoom in while viewing a photograph)

### Page 4 (Background)

What is your child’s date of birth?

What is your child’s sex?

What is your zip code?* *We ask this to have a general idea of our respondents.
